# Immune Infiltration Associated MAN2B1 Is a Novel Prognostic Biomarker for Glioma

**DOI:** 10.3389/fonc.2022.842973

**Published:** 2022-02-02

**Authors:** Xuelei Lin, Hongwei Liu, Hongyu Zhao, Shunjin Xia, Yueshuo Li, Chaoqian Wang, Qi Huang, Siyi Wanggou, Xuejun Li

**Affiliations:** ^1^ Department of Neurosurgery, Xiangya Hospital, Central South University, Changsha, China; ^2^ Hunan International Scientific and Technological Cooperation Base of BrainTumor Research, Xiangya Hospital, Central South University, Changsha, China

**Keywords:** glioma, MAN2B1, immune infiltrates cells, prognosis, tumor-associated macrophage

## Abstract

Mannosidase Alpha Class 2B Member 1 (MAN2B1) gene encodes lysosomal alpha-d-mannosidase involved in the ordered degradation of N-linked glycoproteins. Alteration in MAN2B1 has been proved to be accountable for several diseases. However, the relationship between MAN2B1 and glioma malignancy remains unclear. In this study, RNA-seq data from The Cancer Genome Atlas and the Chinese Glioma Genome Atlas datasets were analyzed to explore the correlation between MAN2B1 and clinicopathological features, prognosis, and somatic mutations in gliomas. We found that MAN2B1 was elevated in glioma and was correlated with malignant clinical and molecular features. Upregulated expression of MAN2B1 is prognostic for poor outcomes in glioma patients. Different frequencies of somatic mutations were found in gliomas between high and low MAN2B1 expression. Real-time quantitative polymerase chain reaction, western blot, and immunohistochemistry staining from glioma patient samples and cell lines were used to validate bioinformatic findings. Functional enrichment analysis showed that MAN2B1 was involved in immune and inflammation processes. Moreover, MAN2B1 expression was strongly correlated with M2 macrophages and weakly correlated with M1 macrophages. Further analysis confirmed that MAN2B1 was closely associated with the markers of M2 macrophages and tumor-associated macrophages. Taken together, MAN2B1 is a potential prognostic biomarker in glioma and associates with immune infiltration.

## Introduction

Glioma is the most common primary malignant tumor of the central nervous system (CNS) in adults (1). Chemotherapy, radiation therapy, and neurosurgical resection are the most common standard treatments for glioma, while immunotherapy (e.g., PD-1, PD-L1, and CTLA4) and targeted treatment have been increasingly used over the last decade. Glioblastoma multiforme (GBM) is an aggressive type of glioma associated with resistance to treatments and recurrence; patients with GBM usually have a short survival rate. For example, immunotherapies, including anti-PD-1, PD-L1, and CTLA4, have shown poor therapeutic effect in GBM patients (2). Previous studies revealed that tumor-infiltrating immune cells (TIICs), such as tumor-associated macrophages (TAMs), influenced the efficacy of chemotherapy and immunotherapy (3). Macrophage cells can be classified into two M1 and M2 macrophages. The M1 macrophage promotes inflammatory response, while the M2 macrophage shows anti-inflammatory functions (4–6). M2 phenotypic macrophages, predominant TAMs in malignant tumors, promote tumor progress by secreting pro-angiogenic factors and immunosuppressive cytokines (7), while an increased proportion of M2 phenotypic macrophage in TAMs has been associated with poor prognostic factors in glioma (8).

Glycoconjugates (glycoproteins, proteoglycans, and glycolipids), which are an essential part of the extracellular matrix (ECM) and basement membranes (BM), have a crucial role in the prevention of cancers (9). For example, glycolipids and proteoglycans participate in the constructions and physiological functions of neurons and glial cells in brain tissue. In addition, glycoconjugates are involved in cell proliferation, differentiation, cell-cell interaction, and signal transmission (10, 11). Thus, many glycoconjugates have been recommended and approved by FDA as biomarkers to identify a given disease condition and monitor patients undergoing therapy clinically (12). For example, CA 125 antigen is a well-known glycoprotein standard marker for ovarian cancer (13). CA 19-9 is a useful pancreatic cancer biomarker (14), while CA 15-3 is breast cancer, especially for evaluating the recurrence of the disease (15). Moreover, a prostate-specific antigen is used as a marker for screening for prostate cancer patients (16). The degradation of glycoconjugates is associated with essential biological functions. The degradation of the ECM and BM elements by tumor-associated enzymes is an essential step that regulates tumor cells’ biological behavior (invasion, growth, and metastasis). Post-translational modification of glycoprotein sugar chains takes place in the endoplasmic reticulum and Golgi apparatus, while the degradation of the oligosaccharide chains of glycoconjugate mainly occurs in the lysosomes (17). Lysosomal acid hydrolases, such as exoglycosidases, can release monosaccharides moiety from sugar chains during the degradation of glycoconjugates (18).

Mannosidase Alpha Class 2B Member 1(MAN2B1) gene encodes lysosomal enzyme alpha-d-mannosidase (EC 3.2.1.24), a kind of acid exoglycosidase, which can catalyze the hydrolysis of α1,2-, α1,3- and α1,6-mannoside linkages during the ordered degradation of N-linked glycoproteins (18). The lack of lysosomal α-D-mannosidase activity leads to the α-mannosidosis [MIM: 248500], a lysosomal storage disorder found in humans, cattle, and cats, characterized by accumulation of partially degraded oligosaccharides in the lysosomes (19, 20). The different α-D-mannosidase activity levels have been found in different phenotypic leukemic lymphocytes (21). Upregulated levels of α-D-mannosidase were found in peripheral cells from patients with Alzheimer’s disease and have been correlated with Ras oncogene activation (22). Also, a recent study found that the high α-D-mannosidase enzyme activity was associated with the MAN2B1 transcriptional upregulation in the promyelocytic leukemia cell lines (23). However, the relationship between MAN2B1 and glioma malignancy needs to be further understood.

In this study, RNA-seq data from The Cancer Genome Atlas (TCGA) and the Chinese Glioma Genome Atlas (CGGA) datasets were analyzed to explore the correlation between MAN2B1 expression and clinicopathological features, prognosis, and somatic mutations in gliomas. We also investigated the association between MAN2B1 and TIICs.

## Materials and Methods

### Bioinformatic Analysis

**Figure 1:**
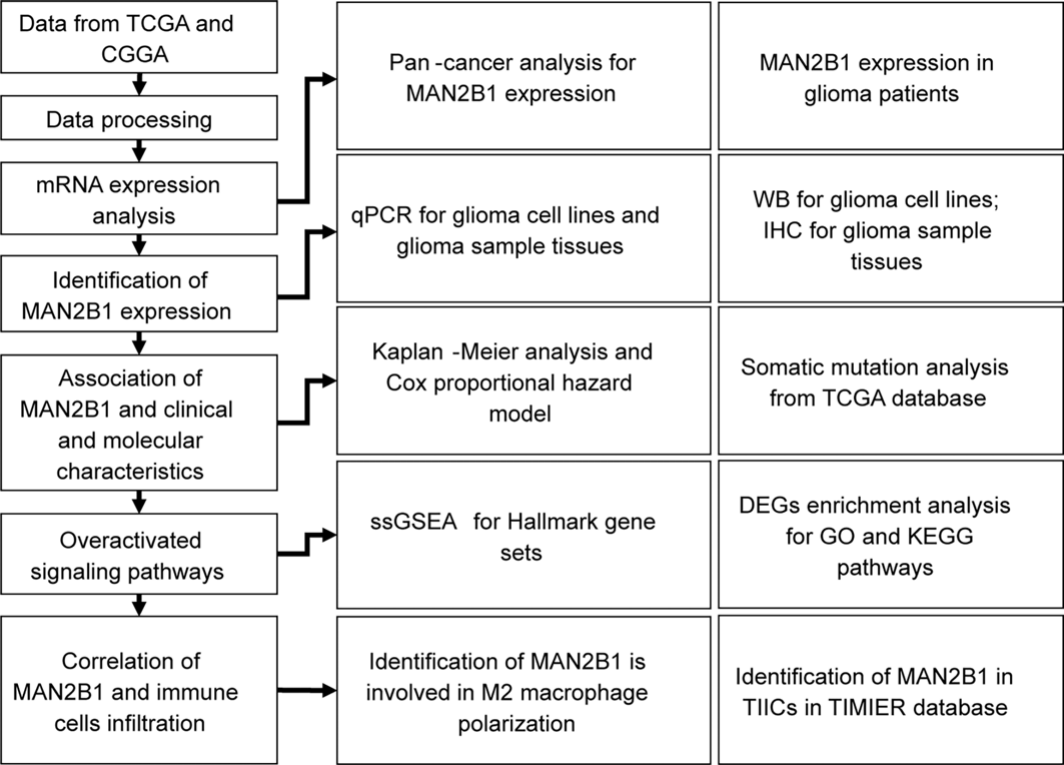
Workflow to show our data collecting and analysis processing.

The workflow of our study is shown in **Figure 1**. The TCGA pan-cancer, GTEx RNA-seq, and their clinical data were downloaded from the UCSC Xena data portal (https://xenabrowser.net/transcripts/). The CGGA RNA-seq and their clinical data were downloaded from http://www.cgga.org.cn. All the RNA-seq expression data were log2 TPM transformed. The ggplot package was applied to visualize data and draw a plot (24). Meanwhile, the limma package identified the differentially expressed genes (DEGs) between the high- and the low-MAN2B1 expressed group. According to the median cutoff, the survival and survminer packages were applied for Kaplan–Meier survival curve. ClusterProfile package was used for conducting Kyoto Encyclopedia of Genes and Genomes (KEGG) and Gene Ontology (GO) enrichment analysis. All the GO terms, including cellular component (CC), biological process (BP) and molecular function (MF) categories were analyzed. A total of 50 hallmark gene sets were obtained from the molecular signature database (MSigDB, http://software.broadinstitute.org/gsea/msigdb). The gene set variation analysis (GSVA) package and its single-sample of Gene Set Enrichment Analysis (ssGSEA) method were used to analyze the GSVA scores of each hallmark gene set for each sample in TCGA (https://portal.gdc.cancer.gov/) and CGGA datasets (25). The GSVA score contributes to the relative enrichment degree of each gene set in each sample. To further evaluate the immune cell proportion in bulk tissues, the CIBERSORT deconvolution algorithm was adopted to estimate the abundances of immune cell in mixed cells. TIMER2.0 (http://timer.cistrome.org) was used to identified the correlation between MAN2B1 and immune cell infiltration in GBM and LGG by the partial Spearman’s correlation (26).

### Data Collection

Human glioma cells U251, U87, A172, T98G, LN229 and HEB (ATCC0459; Shanghai Beinuo Biotechnology Co., Ltd., Shanghai, China) were purchased from Shanghai Cell bank. Cells were cultured in Dulbecco’s Modified Eagle’s Medium (high glucose) supplemented with 10% fetal bovine serum (Bovogen) and 1%penicillin/streptomycin in a humidified atmosphere containing 5%CO_2_/95% air at 37°C. Thirty-six glioma tissue samples were obtained from patients admitted to the Xiangya Hospital of Central South University between January 2008 and November 2020. There were 21 males and 15 female patients aged 15 - 71 years old (median 47.1 years). Tumors were classified according to 2016 WHO classification: 10 WHO II cases, 12 WHO III cases, and 14 WHO IV tumor cases. The patients had no history of radiotherapy or chemotherapy before surgery. All samples were analyzed by immunohistochemistry staining (IHC). Moreover, 8 pairs of glioma sample and normal brain samples were obtained and stored at −80°C for real-time quantitative polymerase chain reaction (RT-qPCR). The Ethics Committee of Xiangya Hospital of Center South University approved this study.

### Real-Time Quantitative Polymerase Chain Reaction

The TotalRNAExtractor (Sangon Biotech, China) was used to extract RNA from glioma cell lines and clinical glioma samples. Then, the RNA was synthesized into cDNAs by the PrimeScript^®^ RT reagent Kit (Takara). RT-qPCR was carried out in a 7500 RealTime PCR System (Applied Biosystems) with SYBR Premix Ex Taq (Takara, Japan). The MAN2B1’s primers were: 5′-GCTTCGAGGGTGAGGACTTC-3′ (forward) and 5′-TCAAGTGGGGAGAGAGGAGG-3′ (reverse). Lyceraldehyde 3-phosphate dehydrogenase (GAPDH) was used as an endogenous control gene (5′-CTCCTGCACCACCAACTGCT-3′ (forward) and 5′-GGGCCATCCACAGTCTTCTG -3′ (reverse)). Finally, Ct values obtained from RT-qPCR were used to calculate the relative levels of MAN2B1 mRNA expression by the 2−ΔΔCt method (27).

### Western Blot

The RIPA lysis solution was added to glioma samples and glioma cell lines for 30 min on ice. After centrifuging the mixture, the protein was collected and then measured using a bicinchoninic acid Protein Assay Kit (Thermo Scientific). The proteins were first separated on a 6% SDS-PAGE and then were transduced to PVDF membranes. Samples were then blocked with 5% BSA in TBST for 1 h at room temperature and then incubated with MAN2B1 antibodies (1:500, abcam, Cambridge, UK) or GAPDH antibodies (1:50000AC033, ABclonal, Wuhan, CHINA) at 37°C overnight. After being washed with TBST three times, PVDF membranes were incubated with peroxidase-conjugated secondary antibodies (1:5000) for 1 h at room temperature. Finally, the results were observed by applying the chemiluminescence detection kit (Sangon, Shanghai, CHINA).

### Immunohistochemistry Staining

Clinical glioma samples were analyzed by IHC with MAN2B1 antibodies (1:50, Thermo Fisher). The staining results were magnified 40 times to observe the IHC staining performance of glioma tissue slides for MAN2B1 by two independent pathologists with the software ImageJ2x. The positive integrated optical density (IOD) and pixel areas of the MAN2B1 positive cells in each slide were observed. The average optical density (AOD) is the value of IOD/AREA, and the higher AOD value indicates a higher level of positive MAN2B1 expression.

### Statistical Analysis

Data analysis and graph generations were completed in R (v4.1.0) and Adobe Illustrator software. Pearson or Spearman correlations were performed for variables. Continuous variables between two groups were evaluated by t-test, otherwise Wilcoxon-test. P <0.05 was considered statistically significant, and all statistical tests were two-sided.

## Results

### MAN2B1 Is Comprehensively Elevated in Human Cancers

**Figure 2:**
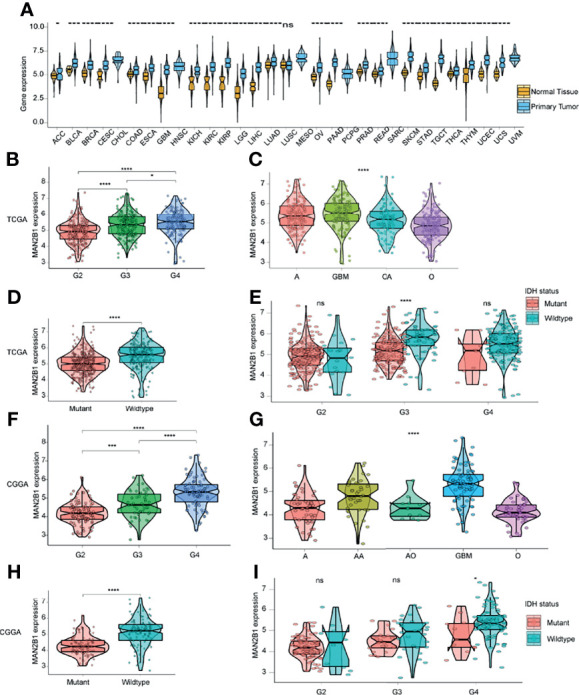
The correlation between MAN2B1 and clinicopathological characteristics in glioma. **(A)** The MAN2B1 mRNA expression in 31 TCGA cancers. **(B, F)** Distinct MAN2B1 expression between different WHO grades in TCGA and CGGA. **(C, G)** Different MAN2B1 expression levels between GBM and astrocytoma, oligoastrocytoma, oligodendroglioma, respectively. **(D, H)** Higher MAN2B1 expression in the IDH wild type compared with IDH mutant gliomas. **(E, I)** The mRNA level of MAN2B1 was higher in IDH wild type gliomas compared with IDH mutant gliomas in each grade. *p < 0.05, **p < 0.01, ***p < 0.001, and ****p < 0.0001. Ns, not statistically significant.

To elucidate the MAN2B1 expression pattern in human cancers, we firstly evaluated MAN2B1 expression by analyzing TCGA pan-cancer RNA-seq data (**Figure 2A**). Elevated MAN2B1 was found in the majority of human cancers, such as bladder urothelial carcinoma (BLCA), breast invasive carcinoma (BRCA), colon adenocarcinoma (COAD). Moreover, MAN2B1 has shown to be over-expressed in both GBM and low-grade gliomas (LGGs) compared with normal brain tissue. These data suggested the role of MAN2B1 in some common pathways in human tumorigenesis.

### MAN2B1 Is Associated With Histopathological and Molecular Features of Glioma

To investigate MAN2B1 expression in glioma, we analyzed the RNA-seq data in TCGA and CCGA databases. The results showed that MAN2B1 was positively correlated with the level of the WHO grade (**Figures 2B, F**). Moreover, according to histopathological classification, higher expression of MAN2B1 was found in GBM than in astrocytoma, oligodendroglioma, oligoastrocytoma, and anaplastic astrocytoma (P<0.05, **Figures 2C, G**). As IDH mutation status is the dominant molecular marker for glioma patients, we analyzed MAN2B1 expression level against IDH mutation status in both pooled and separated WHO grades. We found that MAN2B1 was over-expressed in wild-type IDH gliomas (**Figures 2D, E, H, I**). These results indicated that MAN2B1 might contribute to the malignancy of glioma.

### Verification of MAN2B1 Expression in Glioma

**Figure 3:**
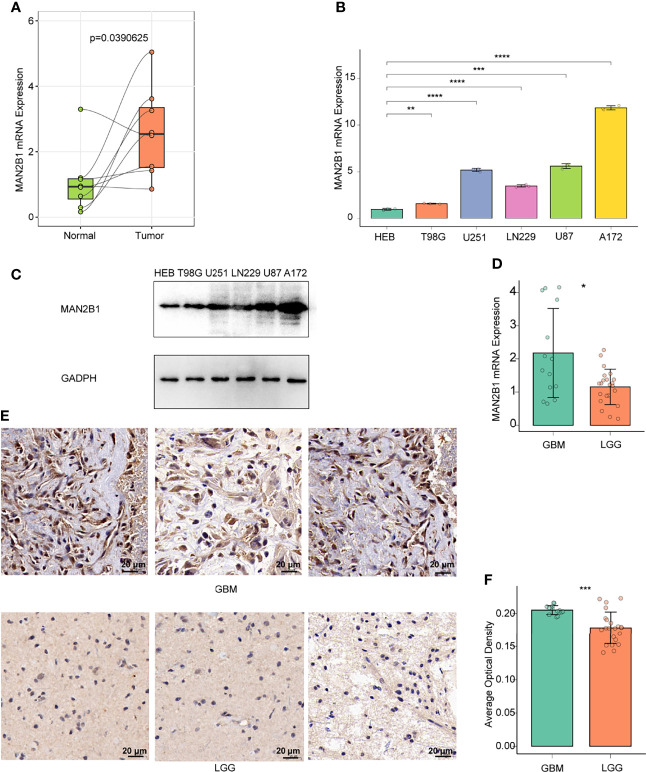
The expression of MAN2B1 was obviously upregulated in glioma tissue. **(A)** RT-qPCR analysis of the different MAN2B1 expression in gliomas and adjacent normal tissues. **(B)** RT-qPCR analysis of the different MAN2B1 expression in glioma cell lines and normal cell lines. **(C)** The expression of MAN2B1 protein in glioma cell lines and normal astrocyte HEB cell line by WB. **(D)** RT-qPCR analysis of the different MAN2B1 protein in GBM and LGG tissues. **(E)** IHC staining of MAN2B1 protein expression in GBM and LGG. **(F)** The average optical density of the positive cells in GBM and LGG tissues. *p < 0.05, **p < 0.01, ***p < 0.001, ****p < 0.0001. Scale bar, 20µm.

To further validate the elevated expression of MAN2B1 in glioma, we tested a series of glioma patient samples from Xiangya Hospital. First of all, we evaluated the mRNA expression of MAN2B1 in 8 pairs of glioma tissue samples (4 GBM and 4 LGG) and adjacent normal brain tissues by RT-qPCR. As shown in **Figure 3A**, higher MAN2B1 mRNA expression was found in glioma tissue than in adjacent normal brain tissues. Then, we tested MAN2B1 expression in GBM and LGG tissues by RT-qPCR. We found that MAN2B1 was upregulated in GBM tissues compared with LGG tissues (**Figure 3D**).

Next, we tested MAN2B1 expression in glioma and normal astrocyte cell lines. Compared with normal astrocyte HEB cell lines, overexpression of MAN2B1 was found in T98G, U251, LN229, U87, and A172 glioma cells (**Figures 3B, C**). To further examine the MAN2B expression in glioma, IHC staining was performed on both GBM and LGG tissues. Compared with LGG tissues, protein expression of MAN2B1 was higher in GBM tissues (**Figures 3E, F**).

Overall, these results confirmed that MAN2B1 expression was upregulated in glioma tissues, and its expression was correlated with glioma WHO grade.

### MAN2B1 Is an Independent Adverse Prognostic Factor in Glioma

**Figure 4:**
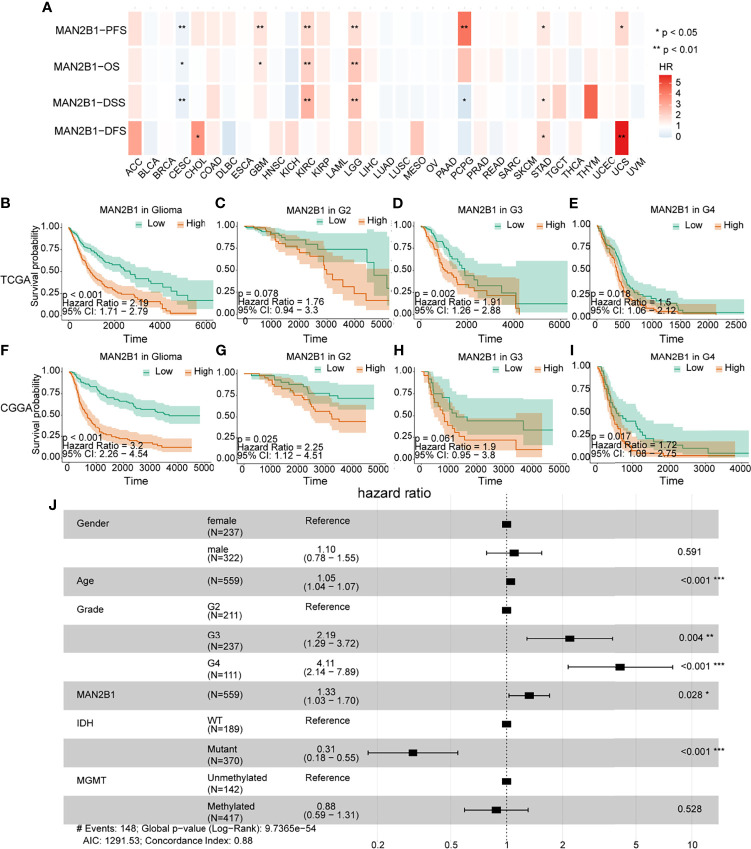
The correlation between clinical outcome and MAN2B1 expression. **(A)** The high mRNA expression of MAN2B1 is associated with poor outcomes in GBM, LGG, CESC, and KIRC. **(B, F)** Poor survival in gliomas with high MAN2B1 expression in both TCGA and CGGA databases. **(C–I)** The prognostic value of MAN2B1 expression in each WHO grade. **(J)** Forest plot shows that upregulated MAN2B1 expression is independent hazard factor for glioma. OS, overall survival; DFS, disease-free survival; HR, hazard ratio; CI, confidence interval. *p < 0.05, **p < 0.01, ***p < 0.001.

Since the MAN2B1 expression was positively correlated with the WHO grade, we tested whether MAN2B1 is a prognostic marker for glioma patients. We first performed a pan-cancer survival analysis of MAN2B1 in the TCGA database. In the TCGA pan-cancer cohort, MAN2B1 resulted as a prognostic factor for progression-free survival (PFS), overall survival (OS), disease-specific survival (DSS), and disease-free survival (DFS) in LGG, GBM, cervical squamous carcinoma (CESC), and kidney renal clear cell carcinoma (KIRC) (**Figure 4A**). Next, we investigated the prognostic value of MAN2B1 in both TCGA and CGGA databases by Kaplan–Meier analysis. The Kaplan-Meier analysis showed that patients with higher MAN2B1 expression had a significantly shorter OS than those with lower MAN2B1 expression (**Figures 4B, F**). Furthermore, following strata analysis, we found that the adverse prognostic value of MAN2B1 was independent to WHO glioma grades (**Figures 4C–E, G–I**).

Next, we performed a multivariate COX regression analysis on MAN2B1 and clinicopathological factors, such as gender, age, WHO grade IDH, and MGMT mutation status. We found that the MAN2B1 expression was an independent prognostic factor for OS and PFS in patients with glioma (high *vs*. low, HR= 1.33, 95%CI= 1.03-1.70, p =0.028, **Figure 4J**). The COX regression analysis on the CGGA database also showed a similar result (HR= 1.48, 95%CI=1.18-1.86, P<0.05, **Supplementary Figure 1**). Overall, our findings indicated that MAN2B1 was an adverse and independent prognostic factor in patients with glioma.

### MAN2B1 Expression Is Associated With Different Patterns of Genomic Alterations

**Figure 5:**
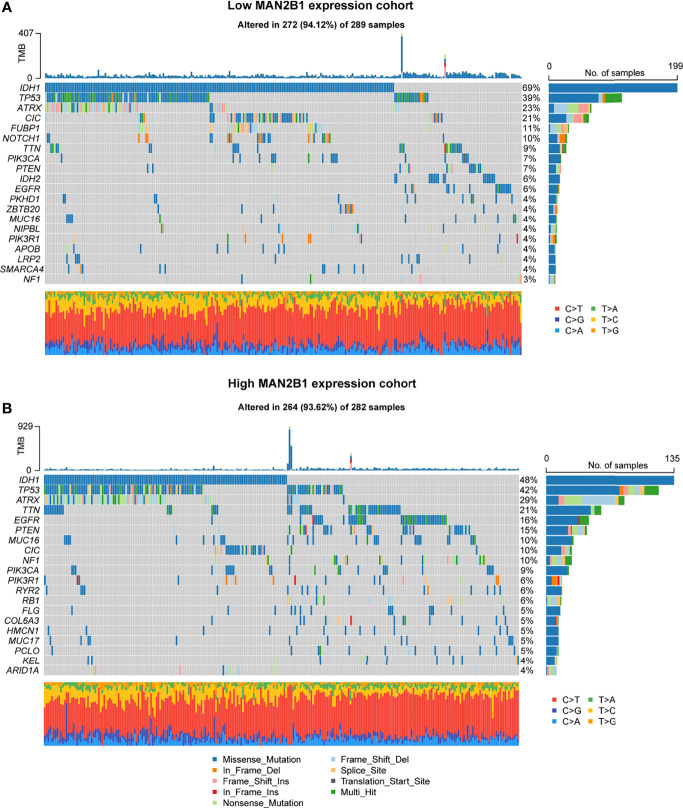
Different genomic profiles associated with MAN2B1 expression. **(A, B)** Distinct somatic mutations were found in gliomas with low MAN2B1 expression **(A)** and in gliomas with high MAN2B1 expression **(B)**.

The somatic mutation data of the patients with glioma obtained from the TCGA were used to analyze the potential molecular mechanisms. The mutation data were divided into the high and low MAN2B1 expression groups, and the mutation frequencies between the two groups were calculated and visualized. As shown in **Figure 5A**, a higher IDH1 mutation frequency was observed in the low MAN2B1 expression group, while the higher TP53, TTN, EGFR, PTEN and NF1 mutation frequencies were enriched in the high MAN2B1 expression group (**Figure 5B**). The results of IDH1 mutation were consistent with our findings on RNA sequences. Additionally, the CIC, FUBP1, and NOTCH1 mutation frequencies also remarkably distinct between the high and low MAN2B1 expression groups (P<0.05).

### MAN2B1 Is Associated With Tumor-Infiltrating Immune Cells in Glioma

**Figure 6:**
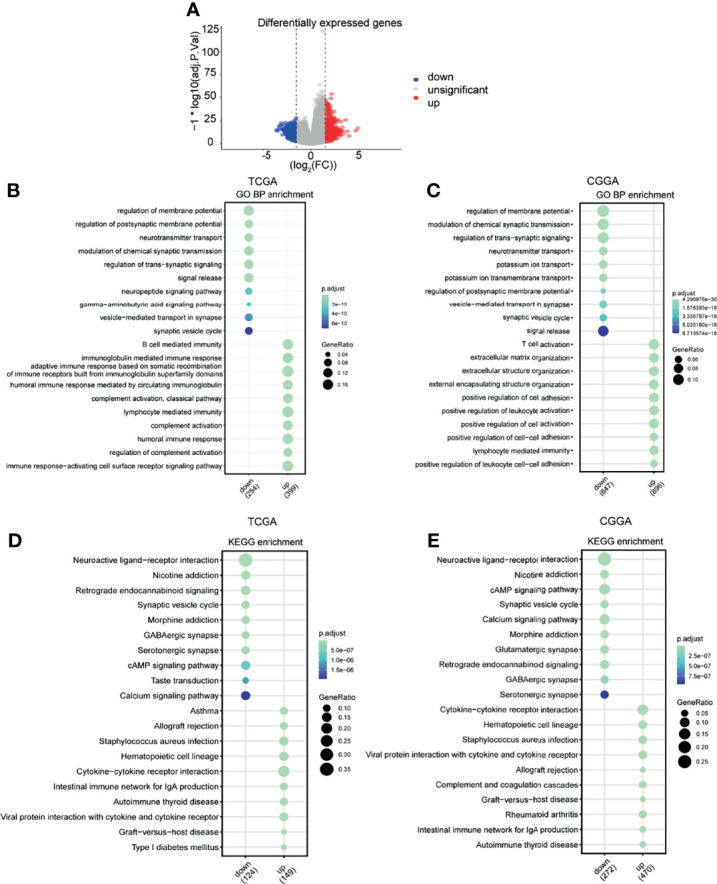
Functional enrichment analysis. **(A)** The DEGs between the high and low MAN2B1 expression groups in the volcano plot. **(B, C)** Enriched GO terms in the BP category showing that the MAN2B1 gene was mostly enriched in the inflammatory response and immune response in TCGA and CGGA datasets. **(D, E)** Enriched KEGG terms showing that the MAN2B1 gene was associated with inflammatory response and immune response in TCGA and CGGA datasets.

Given that MAN2B1 is considered as an independent prognostic factor for glioma patients, we conducted GO and KEGG enrichment analysis on TCGA and CGGA database to explore the molecular function of MAN2B1 in gliomas. The DEGs between the low and high MAN2B1 expression from TCGA and CGGA database were used to predict MAN2B1-related signaling pathways (**Figure 6A**); most enrichened GO terms and KEGG pathways are shown in **Figures 6B–E**. The upregulated genes in the MAN2B1 high group are enriched in immune and inflammation processes, such as humoral immune response, B cell-mediated immunity, immunoglobulin mediated immune response, lymphocyte-mediated immunity, immune response-activating cell surface receptor signaling pathway, and complement activation (**Figures 6B, C**). Besides, these genes are also associated with immune response and ECM, such as positive regulation of cell−cell adhesion, T cell activation, lymphocyte-mediated immunity extracellular matrix organization, extracellular structure organization, and positive regulation of cell adhesion (**Figures 6B, C**). GO terms within the CC and MF categories are shown in **Supplementary Figure 2**. KEGG enrichment analysis suggested that MAN2B1 expression was positively related to cytokine−cytokine receptor interaction, viral protein interaction with cytokine and cytokine receptor, the intestinal immune network for IgA production, and allograft rejection (**Figures 6D, E**).

**Figure 7:**
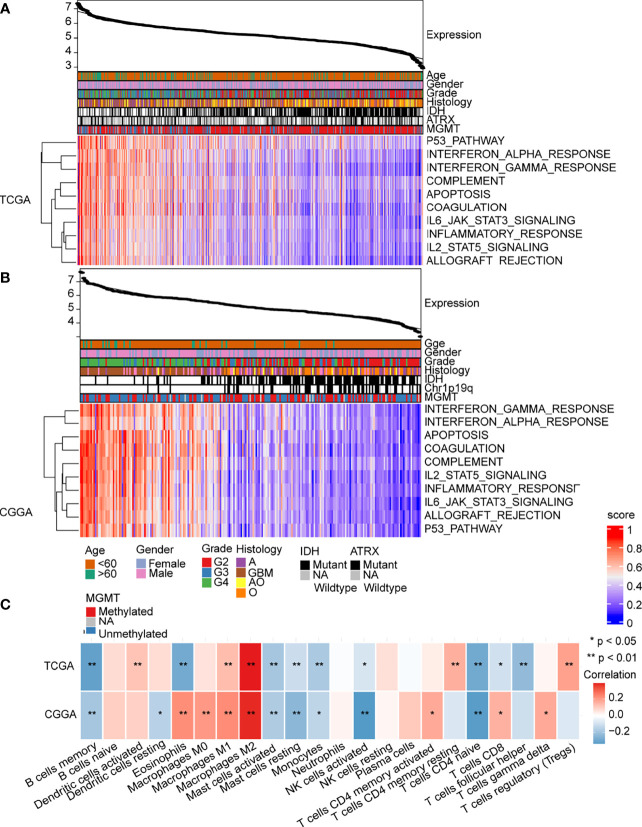
MAN2B1 related immune response and inflammatory activities in gliomas. **(A, B)** Heatmaps showed that the 10 hallmark gene sets were positively correlated with MAN2B1 expression in TCGA and CGGA datasets. **(C)** Correlation between 21 kinds of immune infiltrating cells and MAN2B1 expression.

Based on GO and KEGG enrichment analysis findings, the abnormal expression of MAN2B1 is associated with immune system alterations in glioma. To verify these findings, we performed ssGSEA on hallmark gene sets from MSigDB. Then, Pearson’s correlation analysis was conducted to analyze whether the GSVA score was correlated with the MAN2B1 expression. As shown in **Supplementary Figure 3**, a total of 10 hallmark gene sets, including inflammatory response, interferon-alpha response, interferon-gamma response, complement, IL6_JAK-STAT3 signaling, IL2-STAT5 signaling, P53 pathway, apoptosis, coagulation, and allograft rejection, were obviously correlated with MAN2B1 expression. The heatmap showed that enriched pathways and MAN2B1 expression shared the same trend, thus suggesting that immune-related activation is involved in the glioma progression and influenced the prognosis of glioma patients (**Figures 7A, B**). We further employed the CIBERSORT algorithm to identify the correlations between the total of 22 TIICs with MAN2B1 expression in gliomas. Memory B cells, resting mast cells, activated mast cells, monocytes, activated NK cells, and naive CD4+ T cells were negatively correlated with MAN2B1 expression. On the contrary, M1 and M2 macrophages were positively related to the MAN2B1 expression (**Figure 7C**).

### MAN2B1 Is Correlated With M2 Macrophage Polarization

**Figure 8:**
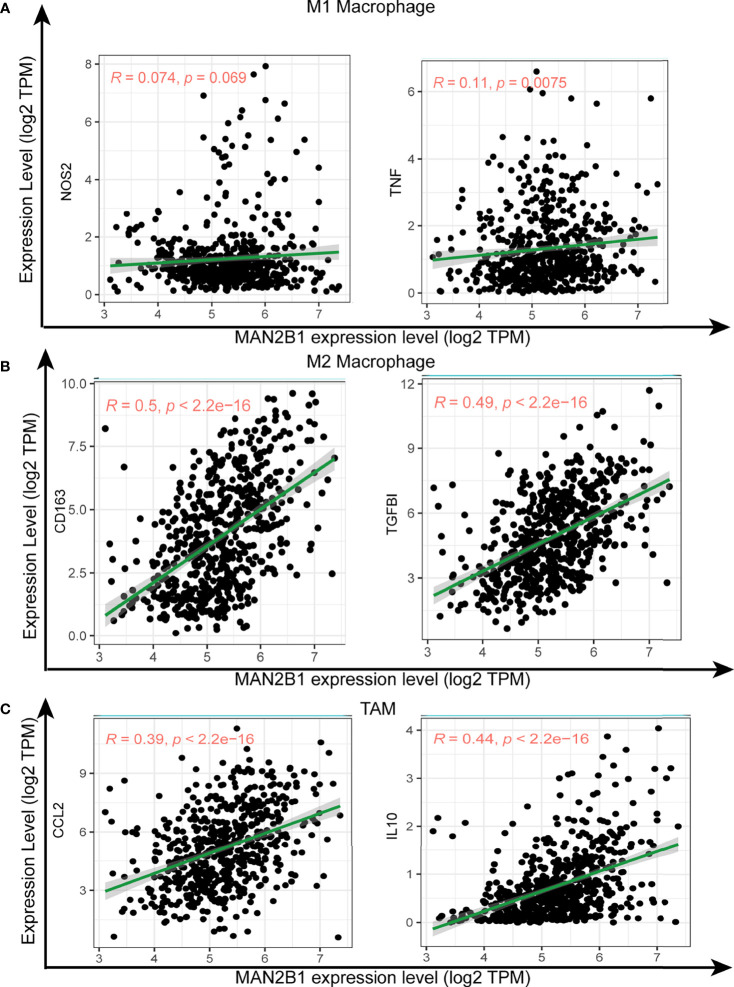
Correlation of MAN2B1 expression with macrophage polarization in glioma. Scatterplots of the correlations between MAN2B1 expression and gene markers of M1 macrophages (NOS2 and TNF) **(A)**, M2 macrophages (CD163 and TGFBI) **(B)**, and TAMs (CCL2, and IL10) **(C)**.

As macrophage polarization contributes to glioma tumorigenesis and MAN2B1 is positively correlated with immune response pathways and immune cells infiltration, we examined whether MAN2B1 is associated with the macrophage polarization. Interestingly, there was a remarkably strong positive correlation between MAN2B1 and M2 macrophages, whereas M1 macrophages showed a moderate positive correlation with MAN2B1 in both TCGA and CGGA database (**Figure 7C**), suggesting that MAN2B1 may affect the polarization of M2 macrophages. The analysis of the correlation between MAN2B1 and the proportion of M1 and M2 macrophages in TIICs from TIMER2 database showed a similar result (**Supplementary Figures 4A, B**). Then the correlation between MAN2B1 and immune checkpoint (PD1 and PD-L1) was analyzed, and we found that there is positive correlation between them (**Supplementary Figures 4C, D**). To further validate the relationship between MAN2B1 and distinct macrophage subtypes, we analyzed the correlation between MAN2B1 and macrophage markers for M1, M2, and TAMs. Interestingly, MAN2B1 showed a significantly strong and positive correlation with M2 macrophage markers, including TGFBI and CD163 (**Figure 8B**). For M1 macrophage markers (NOS2 and TNF), MAN2B1 showed a weak correlation (**Figure 8A**). For TAM markers such as CCL2 and IL10, MAN2B1 showed a moderate correlation (**Figure 8C**). Altogether, these results indicated that high MAN2B1 may promote macrophage polarization, thus contributing to glioma tumorigenesis.

## Discussion

Glioma is the most common and malignant tumor in CNS for adults. Despite the application of T cell immunotherapies and a combination of conventional treatments in the treatment of glioma, patient outcomes are still poor (28). Further understanding of glioma’s tumorigenesis mechanism may improve the clinical outcomes. Mannosidases participate in the biosynthesis and catabolism of N-linked glycoproteins. Lysosomal α-D-mannosidase, one kind of mannosidases, is encoded by the MAN2B1 gene and catalyzes the hydrolysis of α1,2-, α1,3- and α1,6-mannoside linkages during the ordered degradation of N-linked glycoproteins (18). However, so far, the relationship between MAN2B1 protein and glioma remains poorly understood.

In this study, RNA-seq data from TCGA and CGGA datasets were analyzed to explore the correlation of the expression of MAN2B1 and clinicopathological features, prognosis, and somatic mutations in gliomas. The TCGA and CGGA data showed that MAN2B1 expression was significantly upregulated in glioma tissues and was associated with WHO grade, IDH1 mutation status, and histological subgroups of glioma patients. Meanwhile, upregulated expression of MAN2B1 in glioma was validated by RT-qPCR, WB, and IHC staining using clinical glioma samples and glioma cell lines (SHG44, T98G, U251, LN229, U87, and A172). In addition, Kaplan–Meier survival analysis and multivariate COX regression analysis demonstrated that MAN2B1 was an independent prognostic marker for GBM and LGG patients. Similarly, elevated expression of MAN2B1 was also reported in promyelocytic leukemia cell (23). Hence, our results suggest that MAN2B1 is a prognostic factor closely correlated with glioma patients’ outcomes.

The GO terms and KEGG enrichment analysis based on TCGA and CGGA RNA-seq data revealed that upregulated MAN2B1 expression was closely correlated with immune response, such as humoral immune response and complement activation (**Figures 6B, C**). As a result, we believe that MAN2B1 participates in immune response activation of glioma. The GSEA analysis indicated that MAN2B1 was enriched in the inflammatory response, interferon-alpha response, interferon-gamma response, complement, IL6_JAK-STAT3 signaling, IL2-STAT5 signaling, P53 pathway, apoptosis, coagulation, and allograft rejection, which have been all involved in glioma tumorigenesis and malignant development (29, 30). For instance, the abnormal activation of the JAK-STAT signaling pathway leads to carcinogenesis promotion (29). The P53 pathway is considered as a glioma core signaling pathway (30). Collectively, we believe that MAN2B1 influences the prognosis of glioma through immune response and cancer-related hallmark signaling pathways.

GBM displays a high degree of intratumor heterogeneity (31, 32). The tumor microenvironment (TME), including stromal cells and recruited immune cells, is a complex molecular and cellular network that regulates tumor growth (33, 34). Immune infiltrating cells, an important component of the TME, have an important role in cancer malignant progression and immunotherapy response (35). However, there is a lack of evidence on the relationship between MAN2B1 and immune infiltration in gliomas. Our CIBERSORT analysis revealed that MAN2B1 expression was negatively related to memory B cells, monocytes, resting mast cells, activated mast cells, activated NK cells, and naive CD4 T cells, while positively correlated with M1 and M2 macrophages. The dysfunction of NK cells can promote tumor cells proliferation and invasion and, in turn, metastasis (36, 37). It has also been reported that TAMs can express M1 or M2 markers for murine and human cancers, such as glioblastomas (38–40). In breast cancer, head and neck tumors, and pediatric tumors, higher numbers of M2 macrophages have been associated with relatively lower inflammation (41–44). In this study, we found that MAN2B1 expression was positively correlated with the proportion of M2 macrophages, whereas M1 macrophages showed a weaker correlation, suggesting that MAN2B1 was involved in regulating the proportion of TIICs in glioma.

In order to validate whether MAN2B1 expression may influence the macrophage subtypes in glioma, we analyzed the correlation between MAN2B1 and TAMs biomarker genes. As expected, we found that M1 macrophage marker genes, such as NOS2 and TNF, were weakly correlated with MAN2B1 expression (45, 46), whereas M2 macrophage marker genes CD163 and TGFBI were strongly correlated (47, 48). Moreover, TAMs marker genes, including CCL2 and IL10, had moderate correlations with MAN2B1 (49, 50). Our findings suggested that upregulated MAN2B1 expression may promote the polarization from M1 into M2 macrophages and finally into TAMs. These processes are involved in tumor cell angiogenesis, invasion, and metastasis and work as a poor predictive factor for glioma patients (51).

## Conclusion

Our data suggested that elevated expression of MAN2B1 was correlated with clinicopathological features and can be used as a poor predictive factor in glioma patients. Upregulated MAN2B1 expression was associated with TIICs and could promote the polarization of macrophages. Our study revealed that MAN2B1 is a potential prognostic biomarker in glioma and associates with immune infiltrates.

## Data Availability Statement

The original contributions presented in the study are included in the article/**Supplementary Material**. Further inquiries can be directed to the corresponding authors.

## Ethics Statement

The studies involving human participants were reviewed and approved by The Ethics Committee of the Xiangya Hospital Central South University. Written informed consent to participate in this study was provided by the participants’ legal guardian/next of kin.

## Author Contributions

Conception of project: SW and XJL. Experimental design, data acquisition and interpretation: XLL, HL, HZ, SX, YL, CW, and QH. Manuscript writing: XLL, HL, HZ, SW, and XJL. All authors read and critically revised the manuscript for intellectual content and approved the final manuscript.

## Funding

This work was supported by the National Natural Science Foundation of China (for XJL, Grant No. 81770781 and No. 81472594) and the Natural Science Foundation of Hunan Province of China (for SW, Grant No. 2019JJ50978).

## Conflict of Interest

The authors declare that the research was conducted in the absence of any commercial or financial relationships that could be construed as a potential conflict of interest.

## Publisher’s Note

All claims expressed in this article are solely those of the authors and do not necessarily represent those of their affiliated organizations, or those of the publisher, the editors and the reviewers. Any product that may be evaluated in this article, or claim that may be made by its manufacturer, is not guaranteed or endorsed by the publisher.

## Supplementary Material

Supplementary Figure 1 Forest plot for the multivariate Cox proportional hazard regression model. MAN2B1 is an independent prognostic predictor (HR= 1.48, 95%CI=1.18-1.86, P < 0.05). HR, hazard ratio; CI, confidence interval. *p < 0.05, **p < 0.01, ***p < 0.001.

Supplementary Figure 2 Functional enrichment analysis. **(A, B)** Enriched GO terms in CC category in TCGA and CGGA datasets. **(C, D)** Enriched GO terms in MF category in TCGA and CGGA datasets.

Supplementary Figure 3 Correlation between hallmark gene sets and MAN2B1 expression. A total of 10 hallmark gene sets were obviously correlated with MAN2B1 expression in both TCGA and CGGA datasets. *p < 0.05 & |cor | > 0.5, **p < 0.01 & |cor | > 0.5.

Supplementary Figure 4 Correlation between macrophages and MAN2B1 expression. The spearman’s correlation between M1 macrophage infiltration and MAN2B1 expression was weaker, while the M2 macrophage infiltration has a stronger correlation with MAN2B1 both in LGG and GBM **(A, B)**. The correlation between MAN2B1 and immune checkpoint (PD1 and PD-L1) **(C, D)**.
