# Supplementary figures and images for: Immune Infiltration Associated MAN2B1 Is a Novel Prognostic Biomarker for Glioma
Source: Front Oncol. 2022 Feb 2;12:842973. doi: 10.3389/fonc.2022.842973 (PMC8847305; doi:10.3389/fonc.2022.842973)

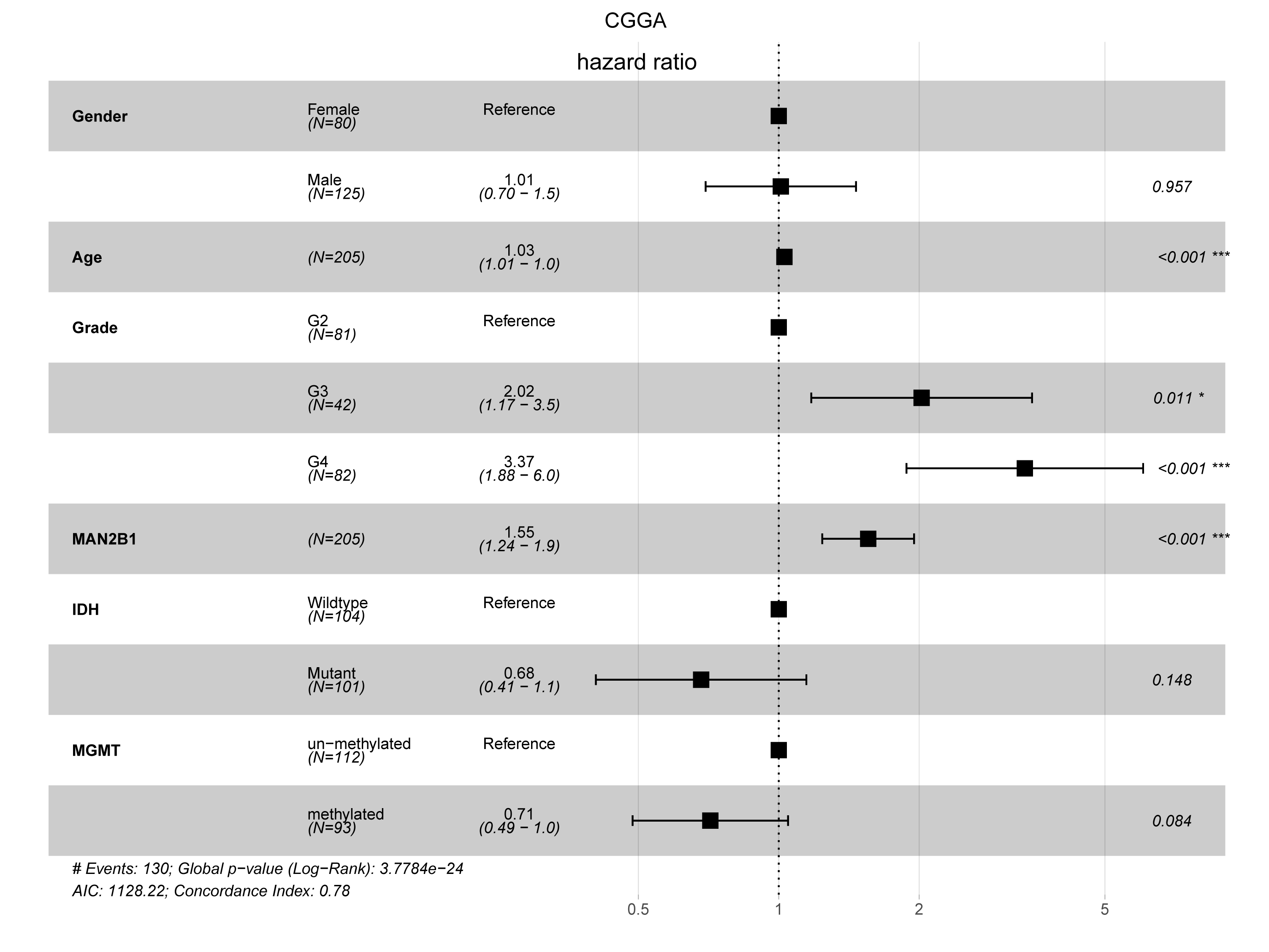

Supplement: Supplementary Figure 1 — Forest plot for the multivariate Cox proportional hazard regression model. MAN2B1 is an independent prognostic predictor (HR= 1.48, 95%CI=1.18-1.86, P < 0.05). HR, hazard ratio; CI, confidence interval. *p < 0.05, **p < 0.01, ***p < 0.001. [file Image_1.tif]

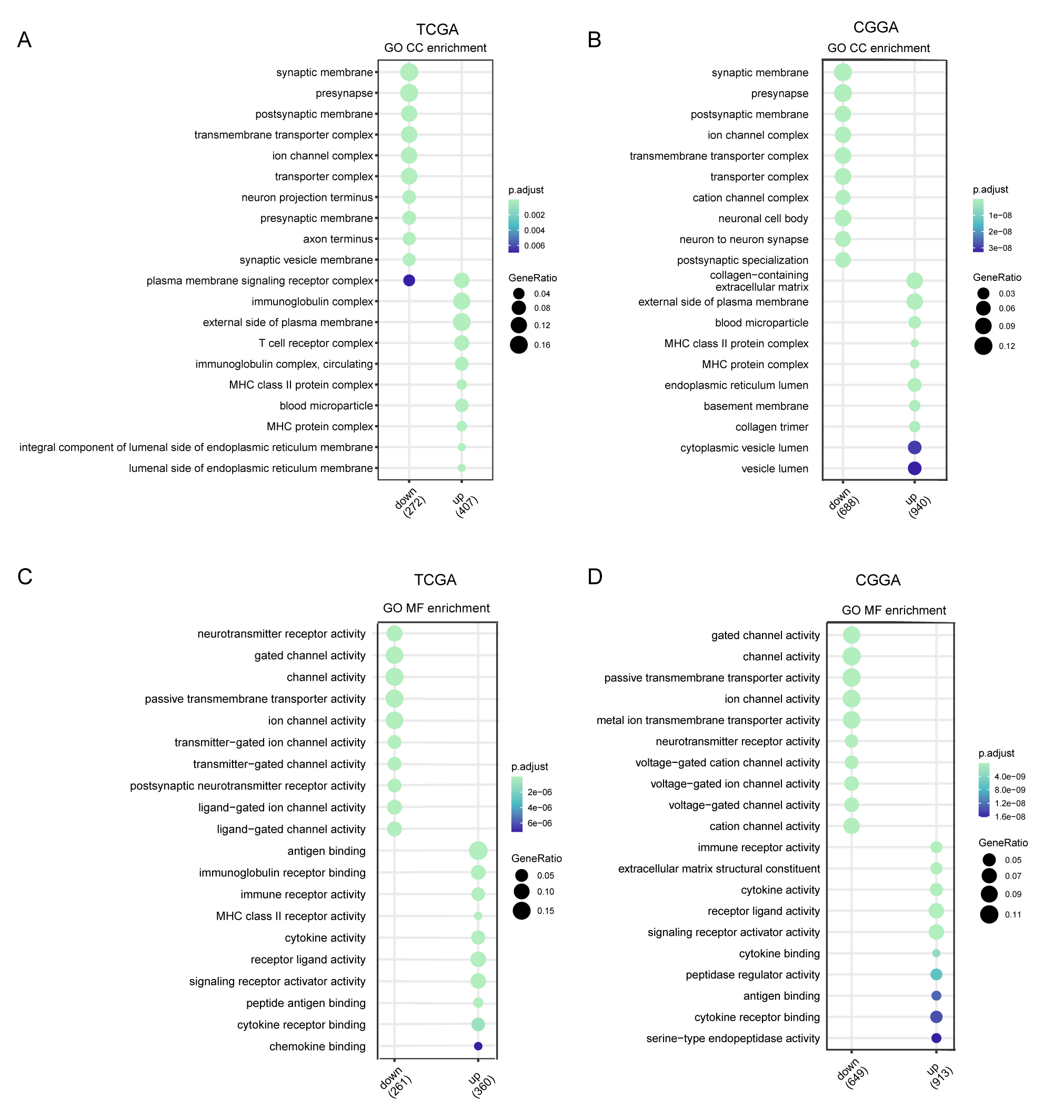

Supplement: Supplementary Figure 2 — Functional enrichment analysis. (A, B) Enriched GO terms in CC category in TCGA and CGGA datasets. (C, D) Enriched GO terms in MF category in TCGA and CGGA datasets. [file Image_2.tif]

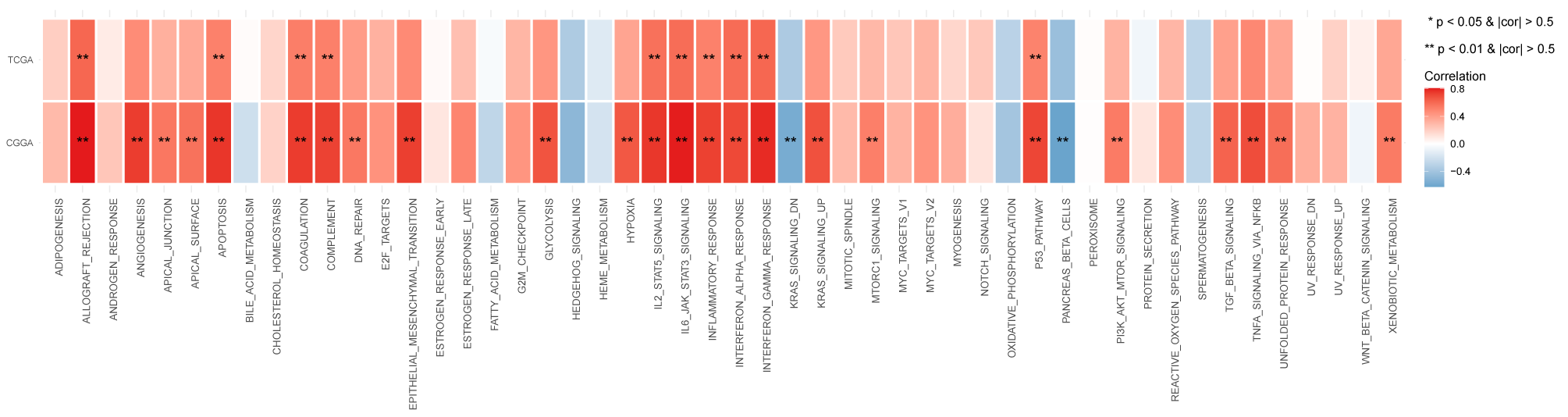

Supplement: Supplementary Figure 3 — Correlation between hallmark gene sets and MAN2B1 expression. A total of 10 hallmark gene sets were obviously correlated with MAN2B1 expression in both TCGA and CGGA datasets. *p < 0.05 & |cor | > 0.5, **p < 0.01 & |cor | > 0.5. [file Image_3.tif]

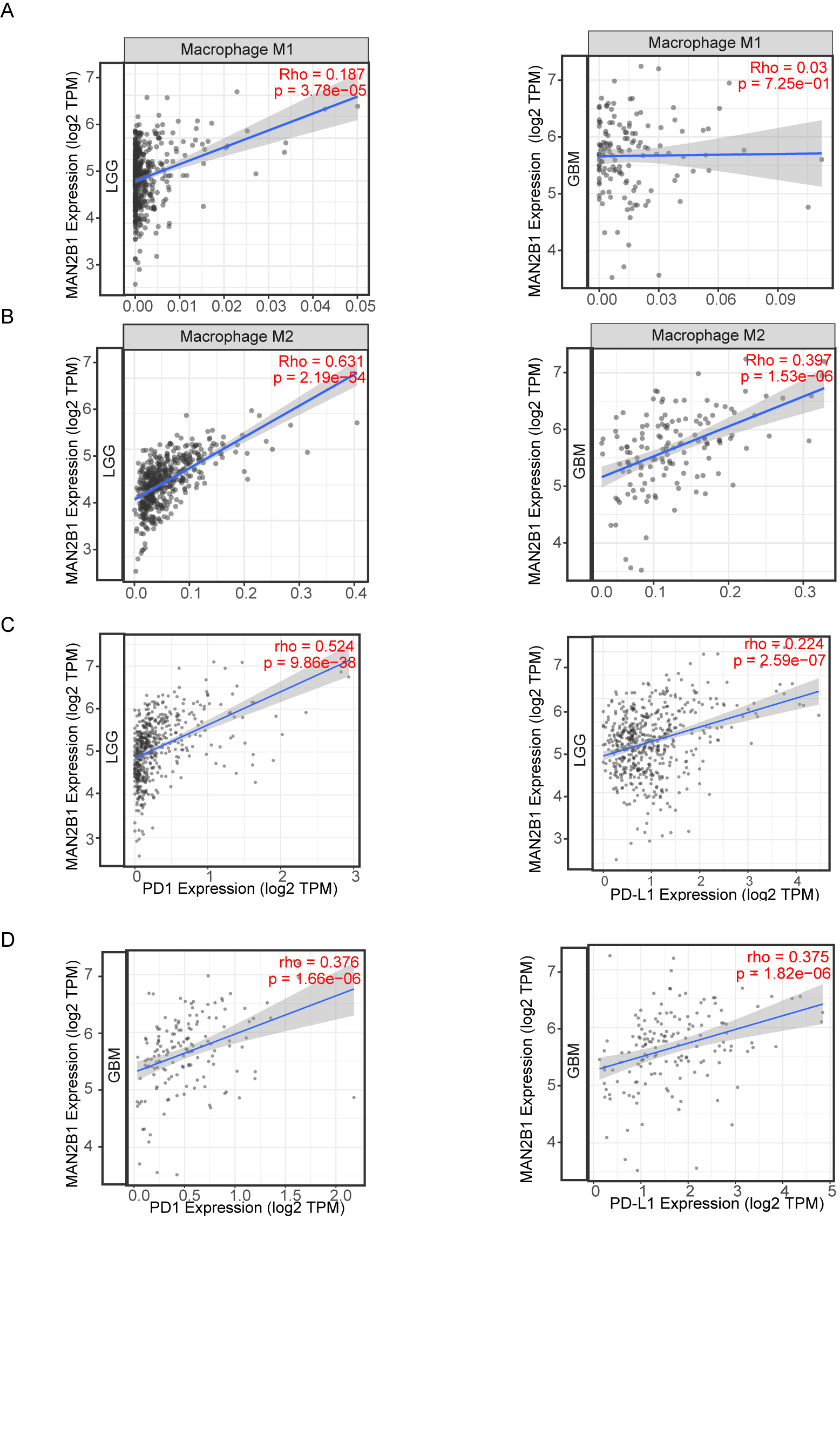

Supplement: Supplementary Figure 4 — Correlation between macrophages and MAN2B1 expression. The spearman’s correlation between M1 macrophage infiltration and MAN2B1 expression was weaker, while the M2 macrophage infiltration has a stronger correlation with MAN2B1 both in LGG and GBM (A, B). The correlation between MAN2B1 and immune checkpoint (PD1 and PD-L1) (C, D). [file Image_4.tif]
